# A Mixed Methods Exploration of the Role of Participation in a Nutrition-Sensitive Agroecology Intervention in Rural Tanzania

**DOI:** 10.1016/j.cdnut.2023.100098

**Published:** 2023-05-13

**Authors:** Marianne Victoria Santoso, Halle Claire Petrie, Rachel Bezner Kerr, Charlotte Lane, Neema Kassim, Haikael Martin, Elias Mtinda, Esther Lupafya, Sera Young

**Affiliations:** 1 Department of Anthropology, Northwestern University, Evanston, IL, United States; 2 Department of Global Development, Cornell University, Ithaca, NY, United States; 3 International Initiative for Impact Evaluation, Washington, DC, United States; 4 Nelson Mandela African Institution of Science and Technology, Arusha, Tanzania; 5 Action Aid Tanzania, Dar es Salaam, Tanzania; 6 Soils, Food and Healthy Communities (SFHC), Ekwendeni, Malawi; 7 Institute for Policy Research, Northwestern University, Evanston, IL, United States

**Keywords:** nutrition-sensitive agriculture, implementation science, participation intensity, participation, process evaluation, agroecology, smallholder farmer

## Abstract

**Background:**

Participation is key to the successful implementation of nutrition-related interventions, but it has been relatively overlooked.

**Objective:**

We sought to describe participation intensity among smallholder farmers in a randomized nutrition-sensitive agroecology study in rural Tanzania. We explored the association between baseline characteristics and overall participation intensity (quantitatively at the individual level and qualitatively at the group level), the association of participation intensity with 2 process indicators, and the association between participation intensity and key study outcomes.

**Methods:**

Data came from 7 rounds of surveys with 295 women and 267 men across 29 months and 2 rounds of semi-structured interviews with the 20 “mentor farmers” who delivered the intervention. Participation intensity was based on the number of months of attendance at village-level project meetings or household visits (range: 0–29). Multivariable models of participation were built.

**Results:**

Women and men participated for 17.5 ± 7.2 and 13.6 ± 8.3 months, respectively. Participation intensity followed 1 latent trajectory: initially low, with a sharp increase after month 7, and plateaued after the first year. At baseline, higher participation intensity was associated with older age, higher education, level of women’s empowerment, being in the middle quintile of wealth, and qualitatively, village residence. Higher participation intensity was associated with 2 process indicators – better recall of topics discussed during meetings and greater knowledge about key agroecological methods. High participation intensity was positively associated with increased use of sustainable agricultural practices among all participants, and among women, with husband’s involvement in household tasks and child’s dietary diversity score.

**Conclusions:**

Participation intensity covaried with key study outcomes, suggesting the value of increased attention to implementation in nutrition-related programs for providing insights into drivers of impact. We hope that investigations of participation, including participation intensity, will become more widespread so that intervention impacts, or lack thereof, can be better understood.

## Introduction

In 2020, ∼9.9% of the world was undernourished, and 118 million more people were facing hunger than the previous year [1]. Nutrition-sensitive interventions attempt to mitigate this burden by addressing the underlying determinants of food insecurity, malnutrition, and the systematic disadvantages that enforce them [2]. Nutrition-sensitive agricultural (NSA) interventions do so through improvement in agriculture [[2], [3], [4]]. NSAs are theorized to improve children’s diets through asset provision as well as behavioral changes and social support from participation intensity [5]. However, although NSA interventions have been found to consistently improve production and dietary diversity, evidence of their impacts on underlying determinants of malnutrition, such as food security, women’s empowerment, and care practices, has been inconsistent [6].

Three recent reviews of NSA impacts proposed 2 explanations for this lack of consistently observed impact [2,6,7]. The first was study design issues such as small sample sizes and inadequate counterfactuals preventing observation of an existing impact. The second was a lack of impact due to suboptimal implementation decisions. For example, short implementation periods of 2 or 3 years were common practice due to limited funding but may not have provided adequate time for an NSA intervention to meaningfully change behavior, farming practices, harvest yield, and ultimately human consumption. Calls have also been made for a closer examination of program impact pathways to understand how NSA interventions shape agricultural and nutritional outcomes [6,7]. In short, many of the explanations posited for why NSA interventions were less impactful than anticipated pertain to implementation science [8]. However, implementation science has been rarely used to analyze reasons for an NSA intervention’s success or failure [9,10].

Participation intensity is a key facet of implementation that has been relatively overlooked in evaluations of NSA interventions. Although it is standard to briefly include a measure of participation when reporting program impact and numerous publications discussed the importance of analyzing participation intensity [[11], [12], [13], [14], [15]], papers analyzing participation intensity and its predictors are rare [16]. Of the 29 papers on NSA interventions identified in a recent systematic review, none analyzed determinants of participation intensity and only 2 analyzed the consequences of participation intensity on program impact [6]. The first analyzed the association between village involvement scores as determined by key informants and child’s anthropometry [17]. The second analyzed the effects of participation (defined by accepting vines, attending one meeting, and/or allowing one home visit by intervention promoters over 3 years) on impact [18]. Although the study ultimately found that participants involved in all 3 tasks experienced better outcomes, this analysis illuminates a limited gradient of participation intensity.

We, therefore, explored participation intensity in the Singida Nutrition and Agroecology Project (SNAP-Tz, https://clinicaltrials.gov/ct2/show/NCT02761876), a participatory nutrition-sensitive agroecology intervention that took place among 591 smallholder farmers with young children in rural Tanzania [19].

Specifically, the objectives of this work were to 1) describe participation intensity, defined as the number of months of participation in project activities out of the 29 months of the active intervention period from August 2016 to January 2018, 2) quantitatively and qualitatively investigate the correlation between baseline characteristics and eventual participation intensity in the intervention, 3) quantitatively examine the association of participation intensity with other process indicators: recall of meeting topics and reported knowledge of key methods, and 4) explore the association between participation intensity and parent study’s outcomes.

## Methods

### Parent project

The Singida Nutrition and Agroecology Project (SNAP-Tz, https://clinicaltrials.gov/ct2/show/NCT02761876) was a randomized effectiveness trial on whether a participatory agroecological peer farmer education intervention could increase sustainable agricultural practices, legume production, food security, infant and young child feeding practices, nutritional status of children and mothers, and gender equity that took place in the rural, semi-arid Singida District of Tanzania from August 2016 to January 2019 [19]. On average, the intervention increased the dietary diversity score by 0.57 food groups in index children. The intervention also improved household food security, usage of a range of sustainable agriculture methods, women’s empowerment, and women’s wellbeing outcomes [19].

Two “mentor farmers” were elected from the participating households by their fellow study participants in each of the 20 villages [19]. After various trainings, mentor farmers led their peers in learning about 3 integrated themes of agroecology, gender equity, and child nutrition through village-level project meetings that took place at least once a month and home visits that took place at least once a quarter [20]. Beyond these minimum requirements, mentor farmers were given autonomy to implement participatory learning as they saw fit, such that the implementation could vary by mentor farmer and village. For example, mentor farmers used various teaching techniques to share information about agroecology, gender equity, and child nutrition, such as creating a finance-related support group, experimenting with botanical pesticides, visiting successful farms, and storytelling to promote gender equity at village meetings. A more detailed description of this study can be found in the article by Santoso et al. (2020) [19].

### Participants

Ten villages out of the 20 selected for the study were randomly assigned to receive immediate intervention, whereas the other 10 received intervention 3 years later and will not be discussed in this article. In each village, 25–30 households identified by village leaders for meeting the following criteria were invited to participate: 1) being food insecure as defined by the community, 2) having a child aged <1 year in January 2016, 3) having access to land and planning to farm in the coming year, 4) intending to reside in that village for the next 3 years, and 5) being interested in experimenting with new farming techniques. Ultimately, we analyzed data from 295 households (295 women and 267 men) from the 10 intervention villages (Supplemental Figure 1).

Written consent to participate in the study and for authorization of all future uses of their data in published research was collected from the participants. Each project participant was invited to participate in the study to improve future interventions similar to their current intervention and was provided with a transportation stipend (∼$2) for each data collection. Data were entered with participant responses anonymized. The list of names and household IDs were saved separately and can only be accessed by principal investigators (RBK, SLY) and study coordinators (MVS, NK, HM). Mentor farmers and participants were informed of survey results after each data collection.

### Data collection and operationalization

#### Survey data

**FIGURE 1:**
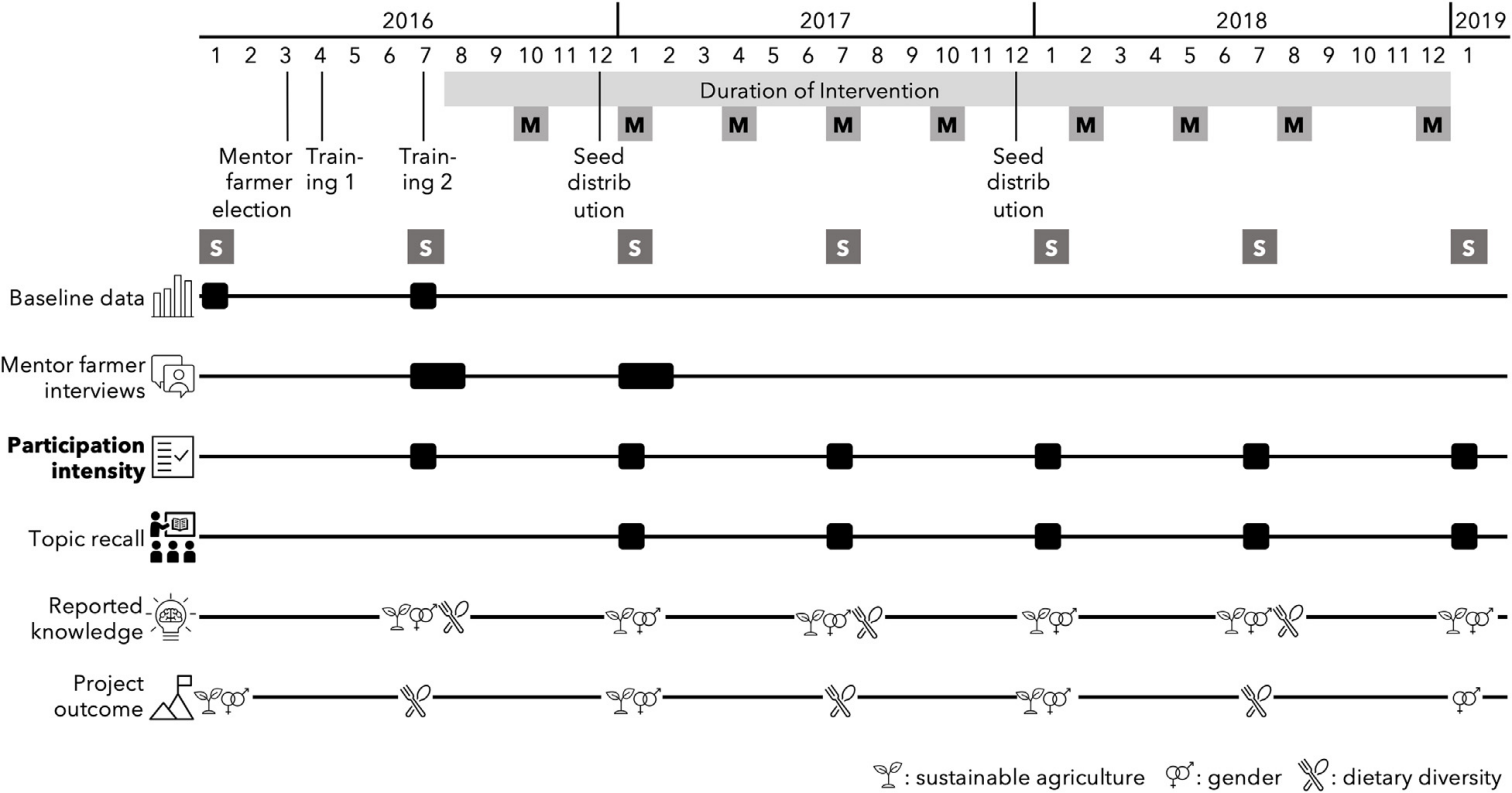
Timeline of Singida Nutrition and Agroecology Project detailing when mentor farmer election, training, seed distribution, mentor farmer quarterly meetings (M), and surveys (S) took place. The timing of data collection of target variables and outcomes is shown; attendance, lessons learned, and knowledge change according to themes of agriculture, gender equity, or nutrition. ∗In July of 2016, only women’s responses for nutrition knowledge change were collected.

Surveys were conducted semi-annually from January 2016 to January 2019 for a total of 7 rounds of surveys. They were timed to correspond to the growing and postharvest seasons (see Figure 1: Study timeline). More information on the survey methodology can be found in the project’s impact evaluation [19].

#### Main key outcome: participation intensity

**FIGURE 2:**
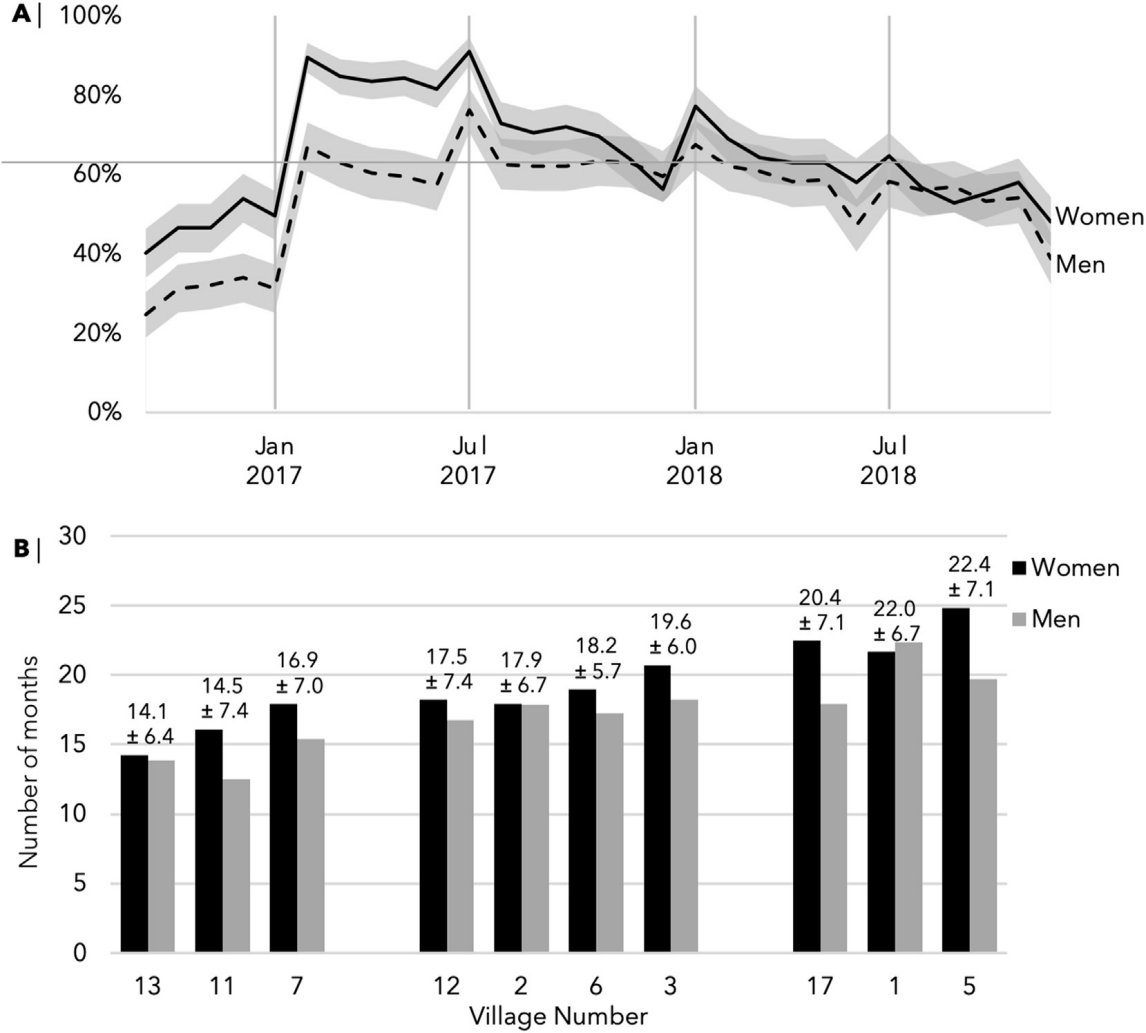
(A) The mean and 95% CI of mean program participation intensity over the course of the Singida Nutrition and Agroecology Project, disaggregated by gender, as indicated by attendance at village-level project meetings and household visits by mentor farmers each month. (B) Average participation intensity (number of months of reported attendance at village-level project meetings and household visits over 29 months) by the intervention village. Villages are grouped based on participation intensity. Villages 5, 1, and 17 had the highest participation intensity, villages 2, 3, and 12 had a moderate participation intensity, and villages 7, 11, and 13 had the lowest.

Participation intensity was measured as the number of months of participation in project activities out of the 29 months of the active intervention period from August 2016 to January 2018. In each survey between January 2017 and January 2019, participants were asked about the number of times they attended village-level project meetings and the number of times they participated in the discussion when mentor farmers visited their households in the previous 6 months (Figure 2). Because the program activities varied widely by village, we standardized responses such that a month was scored as 1 if participants attended ≥1 village-level project meeting or participated in the discussion when mentor farmers visited their households, or 0 if none. This aligned with the minimum number of meetings with participants that mentor farmers were encouraged to have throughout the 29 months of active project implementation period from August 2016 to January 2018 (see Figure 1 for a detailed timeline). Therefore, the total participation intensity continuous variable used in analyses for objective 2 has a range of 0–29. We also created a binary indicator for analyses for objectives 3 and 4 called *high participation intensity*, defined as attending more than half of the meetings in any 6 months (≥4 out of 6 meetings).

#### Baseline predictors of participation intensity

During the baseline survey (January 2016), we collected the sociodemographic data, including marital status, household asset index quantiles (33 items), age, years of education, and religion (Muslim or otherwise) from 295 women and 267 men from participating households (Supplemental Figure 1). We also collected data on food security [21], group membership decision making, perceived social support, and probable depression. We measured overall empowerment with the 5 domains of empowerment score of the Abbreviated Women’s Empowerment in Agriculture Index (A-WEAI) [22] and group membership decision making (1 if the individual had a say, sole or jointly, in the decision to join any village group and 0 otherwise). Adequate social support was defined as scoring a mean of ≥3 on the Perceived Social Support Scale (range, 0-–4) [23]. Probable depression was defined as scoring ≥ 17 on the Center of Epidemiological Studies – Depression Scale (range, 0–60) [24,25]. Because we did not collect data on empowerment, social support, and depression for men in January 2016, we used data from January 2017. Although this is a limitation, this first survey with men was conducted only 6 months after the start of the intervention, and men’s participation in the intervention during this period was very low, so the later survey start was not expected to influence the final measures of participation intensity.

In the surveys between January 2017 and January 2019, we asked participants what they disliked about their participation in the program. Because very few participants mentioned any dislike at any time point and coding of these questions did not yield meaningful aggregation, we simply recorded a binary variable of any mention of dislike of the program by the participant at least once throughout the intervention.

#### Other process indicators

##### Recall of meeting topics

In each survey between January 2017 and January 2019, participants were asked what lessons they learned during village-level project meetings and mentor farmers’ household visits in open-ended questions. We then coded whether each participant mentioned ≥1 of the 3 key impacts that we hoped to affect with the intervention – sustainable agriculture, nutrition, and gender equity – scoring 1 if they did and 0 if they did not for each topic.

##### Reported knowledge of key methods

To assess the participants’ knowledge of sustainable agriculture, we asked them to list any methods they knew to improve soil health in each survey between July 2016 and January 2019. Participants’ answers were coded 1 if they included methods aligned with agroecology, eg, the use of plant residues, composting, mixing soil, rotating crops, and intercropping, and 0 otherwise.

To assess the participants’ knowledge of gender equity, we asked them to describe the characteristics of a good relationship between husband and wife in each survey between July 2016 and January 2019. Participants’ answers were coded as 1 if they included themes on respect, cooperation, and more equitable decision making, and 0 otherwise.

To assess the participants’ knowledge about child nutrition, we asked them to list any methods they know to improve child nutrition in surveys administered in July 2016, July 2017, and July 2018. Participants’ answers were coded as 1 if they mentioned ideas of increasing a child’s dietary diversity or providing a child with legumes or animal-sourced foods, and 0 otherwise.

#### Parent study’s outcomes

We also evaluated how participation intensity covaried with 3 main outcomes of the parent study (Objective 4). The first was the registered primary outcome, the index child’s dietary diversity, as measured by dietary diversity score (when the child was 6 months or older) during the harvest season [26]. These data were collected in the July 2016, July 2017, and July 2018 surveys. The second was the number of sustainable practices households used to improve soil health from the following: 1) using livestock manure, 1) using compost, 3) reincorporation of crop residue into the soil, 4) soil mixing, 5) planting in pits, 6) building ridges, 7) intercropping, 8) adding mulch, and 9) planting vetiver grass in rows [27]. These data were collected by jointly interviewing men and women in January 2016, January 2017, and January 2018 surveys. We also assessed 2 measures of gender equity: women’s participation in decision making and women’s reports of their husbands’ involvement in household tasks from the January 2016, January 2017, January 2018, and January 2019 surveys. Women’s participation in income allocation decision making was indicated by the mean score of 0 if she had no say, 0.5 if jointly decided, and 1 if she had the final say on decisions included in A-WEAI [22]. We measured husbands’ involvement in household tasks by asking women whether their partners had participated in each of the 6 tasks that men rarely do, as identified in an iterative pile-sort activity during the pilot study [28].

#### Social desirability bias

We used the adapted Marlowe–Crowne Social Desirability Scale (MC-SDS) for use in the Tanzanian context during the July 2018 survey [29,30]. The original MC-SDS consisted of 33 true/false statements. The affirmative responses can be summed, with a higher score indicating more bias. For our study, we dropped 5 statements that had been previously deemed inappropriate for use in the African context [30] and 2 additional statements (“I like to gossip at times,” and “I don’t find it particularly difficult to get along with loud-mouthed, obnoxious people”) that were deemed inappropriate during pilot testing in Singida.

#### Semi-structured interviews

Two rounds of semi-structured interviews were conducted with all mentor farmers (*n* = 20, Supplemental Text 1). The first round took place from July to August 2016, before the commencement of the intervention, and the second one took place in November 2016, 2 months after the commencement of the intervention (Figure 2). The interview guide included items about what mentor farmers learned during training, the household selection and election process, experiences with teaching from the curriculum, their knowledge of and personal experience with the themes central to SNAP-Tz, their expectations for the intervention, dynamics of their village group and between mentor farmers, and study management. These interviews were transcribed in Swahili and translated into English.

### Data analysis

To understand participation intensity (Objective 1), we first attempted to identify latent groups in attendance trajectories throughout the intervention using group-based trajectory modeling. Following published methods [31,32], we specified a binomial distribution for each month of attendance and examined models with 1–5 different trajectory groups. We examined quadratic and cubic polynomials to describe the change in severity within each trajectory. We determined the optimal number of trajectory groups to characterize our data and the optimal polynomial form for each group using the Bayesian information criterion (BIC).

To quantitatively investigate baseline predictors of participation intensity (Objective 2), we used multilevel mixed-effects regression to estimate the association between the number of months of attendance with demographics data and variables known to be associated with program participation. The demographic variables that were examined can be seen in Table 1. Because we could not find any analysis of predictors of participation in nutrition-sensitive agriculture interventions, we used those identified in parenting interventions [33,34] and other nutrition programs [[35], [36], [37], [38]]. Covariates in univariate analysis with *P* < 0.2 were included in multivariable linear regressions. Standard errors for all models accounted for clustering at the village level. These analyses were performed using Stata 16 [39].

**TABLE 1:** Baseline predictors of program participation intensity in the Singida Nutrition and Agroecology Project^1^

	Women (*n* = 295)		Men (*n* = 267)	
	Univariable	Multivariable	Univariable	Multivariable
Age (y)	0.15∗ [0.04, 0.26]	0.11∗ [0.05, 0.17]	0.10 [−0.02, 0.21]	0.12∗ [0.01, 0.13]
Education level (y)	0.07 [−0.07, 0.22]	0.40∗ [0.17, 0.62]	−0.16 [−0.63, 0.31]	−0.36∗ [−0.63, −0.42]
Born in current village, binary	−1.18 [−3.05, 0.69]		−0.03 [−0.06, 0.01]	
Muslim religion (ref. any other)	−0.29 [−2.44, 1.87]		3.12∗ [0.35, 5.89]	
Nyaturu ethnic group (ref. any other)	1.79 [−2.61, 6.20]		−3.42 [−11.35, 4.51]	
Adequate social support, binary	0.41 [−1.62, 2.44]		−1.62 [−4.45, 1.22]	
Empowered according A-WEAI, binary	8.77 [−6.13, 23.68]	9.39∗ [7.49, 11.29]	−2.47∗ [−4.52, −0.42]	−2.83∗ [−5.28, −0.38]
Mobility decision making	0.69 [−2.01, 3.39]		−2.33 [−5.76, 1.10]	
Social desirability bias	0.08 [−0.12, 0.28]		−0.05 [−0.36, 0.26]	
Attitude toward IPV	0.30 [−0.05, 0.65]	0.29∗ [0.07, 0.51]	−0.54∗ [−1.03, −0.06]	
Experience with IPV	−0.46 [−2.61, 1.69]		−0.67 [−6.11, 4.77]	
Child’s dietary diversity score (0–7)	1.53∗ [0.66, 2.41]		1.76∗ [0.66, 2.87]	
Marital Status (ref. monogamous married)				
Polygamous married	−1.89 [−5.19, 1.41]		−0.70 [−4.57, 3.17]	
Never married	-4.16 [-8.77, 0.45]		-5.19[-22.61, 12.23]	
Divorced/widowed	0.82 [-3.29, 4.92]			
Dependency ratio	0.78 [−0.20, 1.76]		0.48 [−0.75, 1.70]	
Household size	0.40∗ [0.02, 0.78]		0.51∗ [0.04, 0.98]	
Food insecurity (HFIAS, 0−–27)	0.20∗ [0.05, 0.34]		0.18∗ [0.00, 0.37]	
Household wealth quintile (ref. middle)				
Poorest	−3.52∗ [−5.80, −1.25]	−3.77∗ [−5.80, −1.75]	−3.08∗ [−5.96, −0.20]	−6.53∗ [−10.39, −2.67]
Poorer	2.69∗ [0.33, 5.06]	0.64 [−1.62, 2.90]	1.24 [−1.65, 4.13]	−3.50∗ [−6.51, −0.47]
Richer	−0.30 [−2.40, 1.81]	−0.73 [−3.43, 1.97]	−0.28 [−2.80, 2.23]	−2.98∗ [−4.81, −1.14]
Richest	−0.42 [−2.56, 1.72]	−1.56 [−4.34, 1.22]	−1.46 [−4.10, 1.19]	−2.25 [−4.79, 0.30]
Village (ref. villages 3, 6, 2, and 12)				
Villages 1, 5, and 17	3.74∗ [2.46, 5.02]	3.29∗ [1.53, 5.06]	2.53 [−2.89, 4.99]	2.37 [−.0.41, 5.16]
Villages 7, 11, and 13	−3.07∗ [−4.84, −1.30]	−2.85∗ [−5.47, −0.22]	−3.59∗ [−5.30, −1.90]	−3.58∗ [−5.72, −1.44]
Mention any dislikes about intervention	2.62 [0.51, 4.72]	1.54∗ [0.20, 2.89]	2.85 [0.70, 4.99]	2.54∗ [0.55, 4.52]

Abbreviations: A-WEAI: Abbreviated Women’s Empowerment in Agriculture Index; IPV: intimate partner violence; HFIAS: Household Food Insecurity Access Scale; ref.: referent variable.

∗ *P* < 0.05.

1 The table presents results of multilevel mixed-effects regression to estimate the association between the number of months of attendance (program participation intensity) with demographics data and variables known to be associated with program participation. Significant covariates (*P* < 0.2) were included in multivariable linear regressions. Standard errors for all models accounted for clustering at the village level.

Building on the quantitative analyses and to further investigate predictors of participation (Objective 2), we qualitatively analyzed mentor farmer interview transcripts for nonquantifiable factors of participation intensity. We used the constant comparative method [40] through multiple phases. An initial coding scheme, which focused on events and sentiments that might affect participation intensity, was developed by the first author, second author, and Tanzanian research assistants based on themes that repeatedly appear during interviews. The second author then applied these codes to all transcripts, adding new codes as new themes emerged after discussions with the first author. The unit of analysis was a mention, and thematic salience was determined by the number of mentions of each theme.

To understand the association between participation intensity and recall of meeting topics and knowledge change (Objective 3), we assessed the association between high participation intensity for each 6 months interval with a recall of meeting topics and knowledge level at the end of those 6 months using multilevel, mixed-effects logistic regressions controlling for baseline covariates that were correlated with participation intensity and social desirability bias. For models analyzing knowledge change, we also controlled for knowledge at baseline (July 2016).

To explore the association between participation intensity and the parent study’s outcomes (Objective 4), we assessed the association between high participation intensity for each 6months interval with a recall of meeting topics and knowledge level at the end of those 6 months using multilevel, mixed-effects logistic regressions. We controlled for social desirability bias, outcome measures at baseline, and baseline covariates that were correlated with participation intensity: household wealth quintile, individual’s age, education, 5 domains of empowerment score, and attitudes toward intimate partner violence. For models describing child’s dietary diversity score, we also controlled for the child’s age.

## Results

Most women and men living in intervention villages were monogamously married, Muslim, belonged to the Nyaturu ethnic group, and reported farming as their main occupation (Table 2).

**TABLE 2:** Baseline characteristics of participants in the intervention arm (n = 295 women and children, n = 267 men) of the Singida Nutrition and Agroecology Project.

Household characteristics	
Household size	6.3 ± 2.3
Marital status	
Married (monogamous)	84.1%
Married (polygamous)	7.5%
Separated/divorced/widowed	8.5%
Experiencing moderate or severe food insecurity according to HFIAS	79.4%
Household assets	
Electricity	3.5%
Bicycle	26.1%
Metal roof	16.9%
Women’s characteristics	
Age (y)	29.6 ± 7.8
Years of education	6.0 ± 2.8
Muslim	76.9%
Nyaturu ethnic group	95.9%
Adequate social support	78.4%
Probable depression	41.2%
Men’s characteristics	
Age (y)	37.0 ± 9.7
Years of education	7.5 ± 2.4
Muslim	76.0%
Nyaturu ethnic group	98.3%
Children’s characteristics	
Female	51.5%
Age (mo)	5.8 ± 3.4

Abbreviation: HFIAS, Household Food Insecurity Access Scale.

### Objective 1: describe participation intensity

Trajectory analysis of monthly continuous participation intensity identified only one latent group (Supplemental Figure 2 for the trajectory of the one latent group). Men and women followed a broadly similar pattern based on visual inspection of the graphs (Figure 2A). Participation was initially low, increased sharply after month 7, and leveled off between 55% and 60% from month 13 onward (range: 0–29). Women’s total participation intensity was higher; they attended a mean ± SD of 17.5 ± 7.2 months as opposed to men’s 13.6 ± 8.3 months. Moreover, participation intensity varied by village (Table 1). Villages 5, 1, and 17 had the highest participation intensity, villages 2, 3, and 12 had a moderate participation intensity, and villages 7, 11, and 13 had the lowest (Figure 2B).

### Objective 2a: quantitatively analyze the association between individual baseline characteristics and overall participation intensity in the study

For both men and women, higher participation intensity was associated with older age, higher education, and women being more empowered (according to the A-WEAI; Table 1). The relationship between household wealth and participation intensity differed by gender. Among women, being in the poorest quintile of household wealth was associated with lower participation intensity. Among men, the relationship was U-shaped, with both the poorest quintile and the 2 richer quintiles having lower participation intensity. Village of residence was also associated with participation intensity.

### Objective 2b: qualitatively analyze the association between mentor farmer’s baseline characteristics and village group’s participation intensity in the study

**FIGURE 3:**
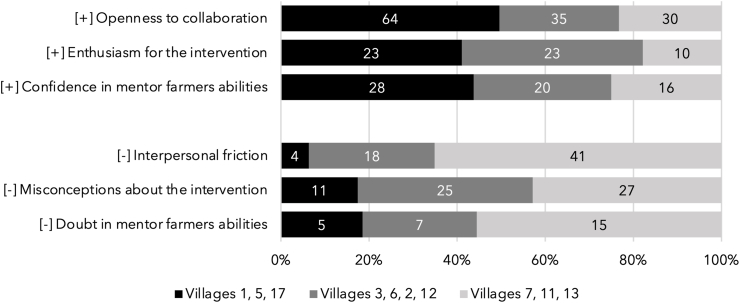
Percentage of the number of mentions of themes that would explain higher or lower program participation intensity identified in mentor farmer interviews according to the attendance tier of participating villages. Villages are grouped based on participation intensity. Villages 5, 1, and 17 had the highest participation intensity, villages 2, 3, and 12 had a moderate participation intensity, and villages 7, 11, and 13 had the lowest (Table 1).

Six major themes of mentor farmers’ attitudes toward the intervention at baseline were identified: 3 that would explain higher program participation intensity (“positive”) and another 3 that would explain lower program participation intensity (“negative”) (Figure 3). The positive themes were collaboration, enthusiasm for the intervention, and confidence in mentor farmers’ abilities. The negative themes were interpersonal friction, misconceptions about the intervention, and doubt about mentor farmers’ abilities.

#### Collaboration

Mentor farmers planned and coordinated various collaborations throughout the intervention. These collaborations included sharing teaching and household visit responsibilities with their partner mentor farmer, visiting and discussing their progress with mentor farmers from other villages, and discussing their progress with and requesting help from village leaders. Some mentor farmers also talked about several ways to encourage active involvement by program participants.

*On the first day, we were together arranging chairs, and we agreed that I lead one lesson on soil, and she would lead the one about nutrition…. She left her work just to visit me so that we could get time to discuss on how we would lead the lessons, so we discussed first before the class [Male mentor farmer, village 12, medium participation intensity].*

*I called [neighboring mentor farmer], and he told me the attendance was also not good there. I asked him what he was doing to make people come to the meeting, and he said that he sometimes postponed, and sometimes they proceeded with just a few [Male mentor farmer, village 5, highest participation intensity].*

*We discussed with the village executive officer [about poor participation intensity in meetings], and he promised to help us to invite all husbands and wives [Female mentor farmer, village 5, highest participation intensity].*

#### Enthusiasm for the intervention

Mentor farmers mentioned their determination to ensure the intervention’s success, expressing belief in the intervention and gratitude for the intervention.

*Because for the coming 3 years, it will not be only 30 people who will benefit, I believe it will be the whole village. [Male mentor farmer, village 17, highest participation intensity]*

#### Confidence in mentor farmer abilities

Mentor farmers expressed confidence in themselves and their peer mentor farmers to teach their peers and function well as leaders.

*I am a community person. I have hospitality; I am mentally fit, and I am also capable of working with this project and cooperating with the people within the project. [Male mentor farmer, village 11, lowest participation intensity]*

*Today, I don’t feel fine, so I ask him to go and visit the households around the village office alone and bring the information to me. He did it well. Also, when we discussed pests, he taught us the way to control pests practically. He used the participants and directed them clearly on what to do and people like that. [name] is good and has experience of work. [Female mentor farmer, village 1, highest participation intensity]*

#### Interpersonal friction

Mentor farmers described tension building and specific instances of conflict that impeded the smooth implementation of the intervention. One example of interpersonal friction was participants’ suspicion of mentor farmers.

*The women who are also in [the intervention] ask us why I did not give anything to them even after I went for training. They think I am hoarding seeds the project gave us. [Male mentor farmer, village 7, lowest participation intensity]*

Another example is the backlash toward the intervention’s messages of gender equity and discussion of family planning. During discussions of these topics in the initial training, one male mentor farmer opposed these messages strongly and had to be replaced for being combative with project staff over the issue. This issue seemed to negatively impact the participation of his village (the 11th village).

*Things that surprise me are when people say this project is to kill children, and they follow [former male mentor farmer] and quit the project [Female mentor farmer, village 11, lowest participation intensity]*

#### Misconception about the intervention

Mentor farmers reported having to manage participants’ misconceptions of the interventions. The most common misconceptions were the participants’ expectation of receiving cash or flour and male participants’ expectation that the program was only for women. Both expectations stem from designs of previous nutrition programs in the area.

*I tell them that this project doesn’t bring you flour or money. It gives you the knowledge so that you can do it yourself so that you will be able to get flour and money on your own. You will be able to succeed in your life, not like every day be given things. [Female mentor farmer, village 11, lowest participation intensity]*

#### Doubt in mentor farmer abilities

This theme includes excerpts of mentor farmers describing a lack of confidence in their abilities, especially when initially elected, and those of their peer mentor farmers. Some of their doubts related to social norms about young people and women, in particular, being less capable.

*They decided to nominate me to be their representative, and I joked with them that I could not do that because, in that group, I was the only one who was young. [Female mentor farmer, village 6, middle participation intensity]*

*[Female mentor farmer] has the challenge to make people understand, and in meetings which we will prepare, I think I will have extra work to explain and educate the community different from her because she is afraid of people. She can speak a few words. [Male mentor farmer, village 3, participation intensity]*

### Objective 3: qualitative examination of the association of participation intensity with process indicators: recall of meeting topics and knowledge change

#### Describing recall of meeting topics and knowledge

**FIGURE 4:**
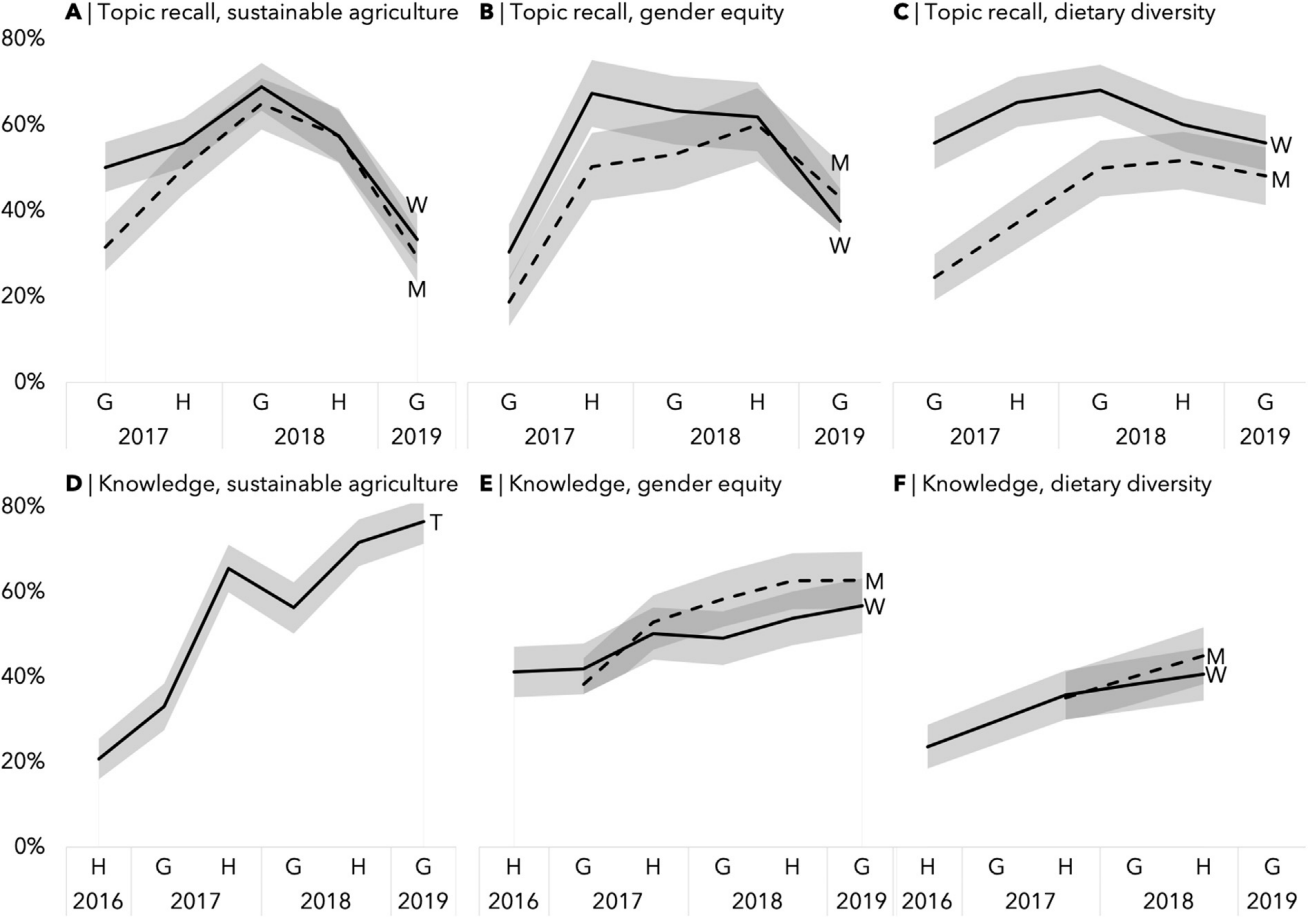
Percentage of men (M, n = 259), women (W, n = 286), or men and women answering together (T, n = 256) who recall discussion of sustainable agriculture (A), gender equity (B), and dietary diversity when asked about topics discussed during village-level project meetings and who answered according to the message intended by intervention about sustainable agriculture (D), gender equity (E), and dietary diversity (F). G indicates data collected during the Growing Season (January and February), whereas H indicates data collected during the harvest season (May, June, and July).

When asked to recall meeting topics, on average, 45.2% of men and 50.8% of women recalled themes of sustainable agriculture (Figure 4A), 49.2% of men and 46.8% of women recalled gender equity (Figure 4B), and 59.1% of men and 70.4% of women recalled child nutrition (Figure 4C). There seemed to be an increase in participants’ recall of relevant topics in the first year of implementation and a decrease thereafter. This trend was most pronounced in the measure of sustainable agriculture. Men initially mostly recalled topics of agriculture in the first year and were more likely to recall discussing gender equity and nutrition in the second year.

When analyzing participants’ knowledge throughout the intervention, on average, 44.4% of households described practices aligned with sustainable agriculture to improve soil health (Figure 4D), 50.3% of men and 47.1% of women described respect, cooperation, and equal decision making when asked about characteristics of a good relationship (Figure 4E), and 43.1% of men and 32.5% of women described dietary diversity when asked about ways to improve child nutrition (Figure 4F). There was a trend of improvement in knowledge across all 3 topics, although the knowledge improvement in dietary diversity and gender equity was much smaller than knowledge improvement in sustainable agriculture.

#### Association between participation intensity and recall of meeting topics and knowledge change

**FIGURE 5:**
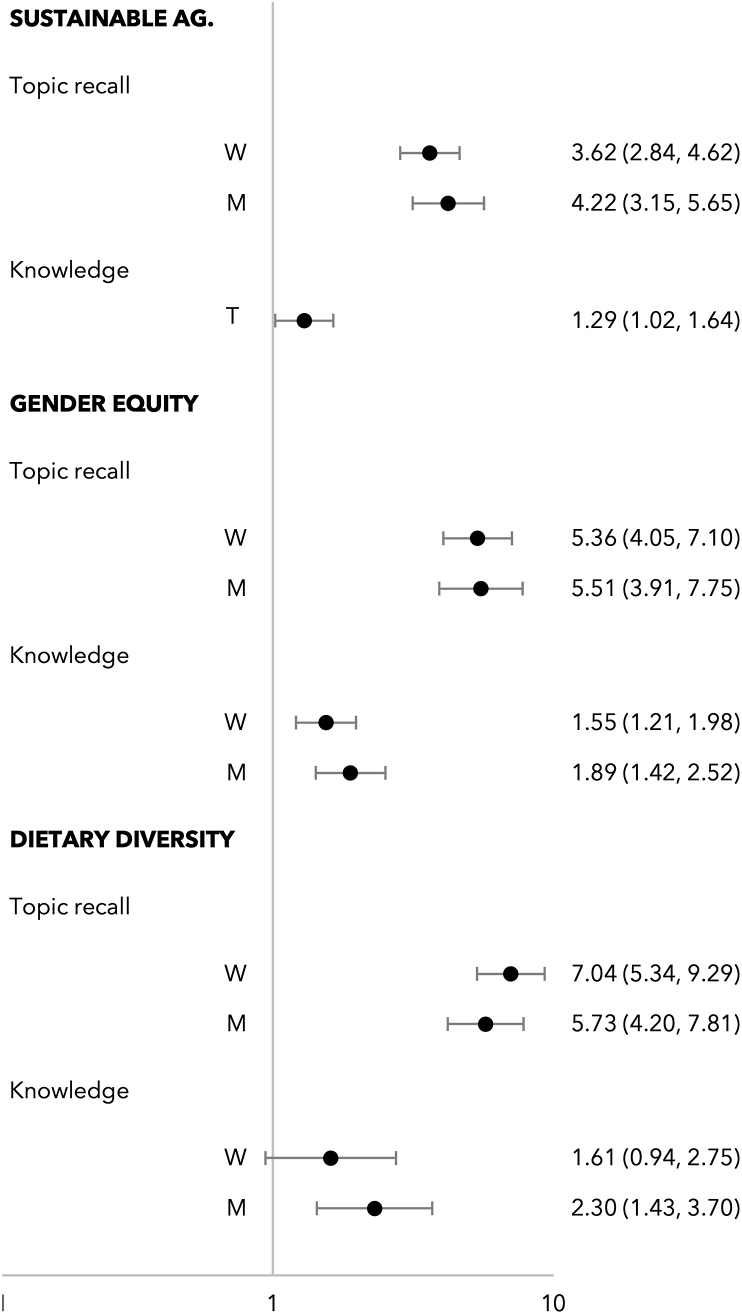
Odds ratio of high participation intensity (attending 4 or more months in any 6 months) on the recall of meeting topics (“topic recall”) and knowledge change (“knowledge”) in Singida Nutrition and Agroecology Project at the end of those 6 months, by gender. W indicates women (n = 286), M indicates men (n = 259), and T indicates men and women interviewed together (n = 259). Models were analyzed separately for each sample. Estimates controlled for social desirability bias and baseline covariates that were correlated with participation intensity: household wealth quintile, individual’s age, education, 5 domains of empowerment score, and attitudes toward intimate partner violence. For models on knowledge change, we also controlled for baseline knowledge on the topic.

High participation intensity was associated with the recall of meeting topics and knowledge change in most topics for both men and women (Figure 5). The only exception was that a high participation intensity was not statistically significantly associated with knowledge change in a child’s diet among female participants (OR = 1.61, 95% CI: 0.94, 2.75). For all 3 themes, the association between participation intensity with the recall of meeting topics was much larger in magnitude than with knowledge change.

### Objective 4: explore the association between participation intensity and key study outcomes

**FIGURE 6:**
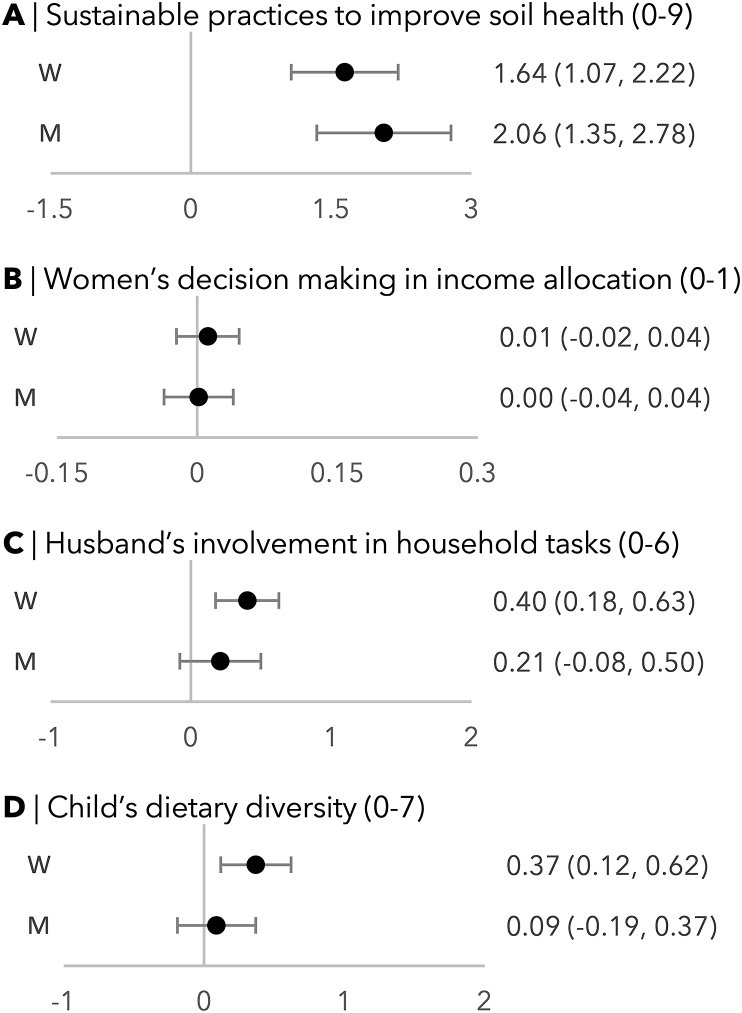
Beta coefficients of the association between participation intensity on various study outcomes of the Singida Nutrition and Agroecology Project by gender. W indicates women (n = 286) and M indicates men (n = 259). We controlled for social desirability bias, outcome measures at baseline, and baseline covariates that were correlated with participation intensity: household wealth quintile, individual’s age, education, 5 domains of empowerment score, and attitudes toward intimate partner violence. For models for child’s dietary diversity score, we also controlled for the child’s age.

High participation intensity was positively associated with the number of sustainable practices to improve soil health used by households throughout the intervention, as reported by both men and women (Figure 6A). Among women, high participation intensity was associated with the husband’s involvement in household tasks (Figure 6C) but not associated with any changes in women’s decision making in income allocation (Figure 6B). Among men, there was no association between participation intensity and either husband’s involvement in household tasks or women’s decision making. High participation intensity was correlated with child’s dietary diversity in women but not in men (Figure 6D).

## Discussion

We explored program participation intensity in the implementation of a participatory nutrition-sensitive agroecology intervention in rural Tanzania. We found that participation intensity followed 1 latent trajectory: it was initially low, increased sharply after month 7, and plateaued between 55% and 60% after the first year of implementation (Figure 2A). Descriptively, women had higher attendance overall, although the difference was much more pronounced during the first year of implementation. Plateauing after the first year was also observed in recall of meeting topics and knowledge change among the participants (Figure 4).

To our second objective, higher participation intensity was associated with older age, higher education, women being empowered according to the A-WEAI, and being in the middle quintile of wealth at baseline (Table 1). Participation also varied between villages (Figure 2B; Table 1). Analysis of semi-structured interviews with mentor farmers revealed positive (collaboration, enthusiasm for the intervention, and confidence in own and other mentor farmer’s abilities) and negative (interpersonal friction, misconception about the intervention, and doubt in own and other mentor farmer’s abilities) themes that might explain these intervillage variations (Figure 3).

To our third objective, high participation intensity was associated with greater information recall and knowledge change about most topics, the 2 process indicators we evaluated (Figure 5). As for our fourth objective, we found that greater participation intensity was associated with the implementation of a greater number of sustainable agricultural practices among both men and women (Figure 6). High participation intensity by women but not men was associated with husband’s involvement in household tasks and child’s dietary diversity score.

Participation intensity in this intervention (55%–60% after the first year of implementation) was on the high end of values reported by other nutrition-sensitive agriculture interventions. Most nutrition-sensitive agriculture interventions reported between 22% and 29% of participants in the intervention group ever attending group meetings [[40], [41], [42], [43], [44]]. Two interventions reported a higher level of participation: the Suaahara program in Nepal found that 55.3% of their homestead food production beneficiaries participated in a group meeting in the season before the survey [45], and 72.4% of women of school children participated in a school garden intervention in the Philippines had high attendance in nutrition education sessions, ie, attended all 6 [46].

The flat trajectory in program participation helps to explain why the impact of this intervention in month 30 of implementation seemed similar to the impact in month 18 [19]. There are at least 2 possible explanations for the plateauing participation intensity in this intervention. The first is that by the start of the second year of implementation, participants had enough information about what the intervention could offer them and had already made their decision about whether to participate in village-level project meetings and to engage with mentor farmers during household visits. The second is related to the reduced attention from project management. At the end of the first year of implementation, the project manager was replaced due to restructuring within the implementing organizational partner. The new project manager brought more qualifications and decision making power to the organization but had to manage a larger number of program portfolios.

The analyses of baseline predictors highlight one of the challenges of implementing NSA interventions: those who need the program the most participated less, likely due to time and resource constraints. In this study, this group included younger parents, those with lower levels of education, women who were less empowered, and those from poorer households. This impact of structural constraints on participation intensity is similar to what has been reported in parenting and nutrition studies [[33], [34], [35], [36], [37]]. Surprisingly, 3 common predictors of program participation intensity—adequate social support [38], parental probable depression [33], and household food insecurity [34]—were not found to be associated with program participation intensity after controlling for the village of residence. This might be due to the lack of within-village variation of these indicators at baseline. For example, 79.4% of households experienced moderate and severe food insecurity, and 78.4% of women had adequate social support at baseline.

We also found high intervillage variation in attendance, which highlights the role of mentor farmers’ attitudes and performance in participants’ overall participation intensity (cf. Figure 2B and Table 1). Because we only have data for 20 mentor farmers in 10 villages, we do not have an adequate sample size to explore this relationship quantitatively. Qualitative analysis of interviews with mentor farmers at baseline, however, revealed that mentor farmers’ attitudes and experiences early in the intervention could be a good indicator for the rate of participation in that village throughout the intervention [8,47]. Confidence (and doubt) in their own and other mentor farmer’s abilities were also found to be important in a process evaluation of the homestead food production program in Cambodia [48]. The misunderstanding that nutrition programs were solely for women was also found by a nutrition-sensitive agroecology program in Malawi [49] and may explain women’s overall higher participation intensity, recall of meeting topics, and knowledge change. It is worth noting that this trend was found despite efforts by mentor farmers and implementing partners to counteract it after the first 6 months of intervention. Clarifying misconceptions about the intervention (such as not giving out flour or money) might not increase participation if the intervention does not align with participants’ interests.

Participation intensity was positively associated with the 2 process indicators: recall of meeting topics and knowledge change. Although this is an expected result, there has been little empirical evidence to support it in nutrition-sensitive agriculture interventions [6,11,50]. One exception is a nutrition-sensitive program in the Philippines that found that high attendance in nutrition education sessions was associated with increased knowledge of nutrition [46]. The smaller magnitude of association between high participation intensity and knowledge change compared with that with the recall of meeting topics might be due to the open-ended nature of the questions asked. For example, some people might agree that equal decision making and task division are important but simply forget to mention them when asked about what makes a good household.

The mixed association between high participation intensity and parent’s study outcomes was somewhat expected. A positive association between high participation intensity and sustainable agricultural practices is consistent with previous findings that behavior change in agricultural practices is usually the first and easiest to achieve in an NSA intervention [2]. The difference in results for the 2 gender equity outcomes can be explained by information found in interviews with mentor farmers later in the intervention as well as notes from project meetings: mentor farmers decided to focus their gender equity messaging on household tasks instead of decision making [19,22]. Surprisingly, men’s participation intensity was not associated with any gender equity outcomes and child’s dietary diversity. This is somewhat of a contrast to the common wisdom stressing the importance of increasing men’s participation intensity in NSAs to ensure an impact on gender equity and nutrition outcomes. One possible explanation for this unexpected finding is how most men’s interest in the project was focused on the agricultural aspect of the project [19,22]. It might also be an important reminder that a project’s participation does not guarantee attitude change. Regardless, this finding suggests the need for further study to explain the dynamics of household decision making about children’s diets and what influences their change.

Our study had several strengths, including a detailed participation intensity indicator, use of myriad process indicators, and use of quantitative and qualitative methods in answering the same research question. Limitations included missing observations, not collecting men’s wellbeing data at baseline and reliance on self-reports. For example, due to some issues with survey logistics, ∼18% of men in the intervention group surveyed in January 2017 were not asked about their project participation. These missing data were not expected to be associated with participation intensity in those first 6 months. Furthermore, because we did not collect data on empowerment, social support, and depression for men in January 2016, we relied on data from January 2017. We believe that the January 2017 data were somewhat representative of baseline characteristics because the data used came from 6 months after the intervention started, and men’s participation in the intervention during this period was low. Finally, we heavily relied on self-reports, which makes our results vulnerable to social desirability bias. We took precautions to address this by avoiding leading questions, using different personnel for enumeration and intervention implementation, and assurance of “no wrong answers” throughout the survey. Furthermore, we measured social desirability bias and found it to be low [19]. Furthermore, we included it in the regressions in all models.

Our analysis of participation intensity revealed various important insights for understanding the impacts of this NSA intervention, such as a possible explanation for why the impact of this intervention in month 30 of implementation seemed similar to the impact in month 18, the importance of paying special attention to younger, less empowered, and poorer households in NSAs, key characteristics in mentor farmers to ensure higher overall participation intensity, and the need for further study on men’s participation and changing attitudes toward gender equity in an NSA. Taken together, this work not only reinforces the need for increased attention to implementation science but also suggests how participation can be empirically studied, contributing to a holistic mixed methods framework that can be referred to by future programs and participation-oriented analyses. We hope that such analyses will become more widespread to help better understand the nature of the dynamics within a given intervention, ultimately improving results for both the implementers and target populations.

## Funding

This study was funded by the Collaborative Crop Research Program of the McKnight Foundation and Atkinson Center for a Sustainable Future of Cornell University. SLY was supported by the National Institutes of Health (K01 MH098902). This work was funded by the Innovative Methods and Metrics for Agriculture and Nutrition Action (IMMANA) program, led by the London School of Hygiene & Tropical Medicine (LSHTM). IMMANA was cofunded by UK Foreign Commonwealth and Development Office (FCDO), grant number 300654 and by the Bill & Melinda Gates Foundation INV-002962/OPP1211308. Under the grant conditions of the Foundation, a Creative Commons Attribution 4.0 Generic License has already been assigned to the Author Accepted Manuscript version that might arise from this submission. The study resulting in this publication was also assisted by a grant from the Undergraduate Research Grant Program which is administered by Northwestern University's Office of Undergraduate Research. However, the conclusions, opinions, and other statements in this publication [or presentation] are the authors’ and not necessarily those of the sponsoring institution.

## Author disclosures

All authors report no conflicts of interest.

## Data availability

Data described in the manuscript, code book, and analytic code will be made available upon reasonable request.

## Appendix A Supplementary data

Multimedia component1 Multimedia component1
