# Supplementary material for: A Mixed Methods Exploration of the Role of Participation in a Nutrition-Sensitive Agroecology Intervention in Rural Tanzania
Source: Curr Dev Nutr. 2023 May 13;7(6):100098. doi: 10.1016/j.cdnut.2023.100098 (PMC10314235; doi:10.1016/j.cdnut.2023.100098)
Supplement: Multimedia component1 [file mmc1.pdf]

Supplementary Data

Title: A mixed-methods exploration of the role of participation in a nutrition-sensitive agroecology intervention in rural Tanzania

First author: Marianne V. Santoso

Online Supplemental Material

Supplemental Text 1: Mentor Farmer Interview Guide…………………………………………………………..2

Supplemental Text 1.1: Mentor Farmer Interview Guide, Round 1…….…………………………………...2

Supplemental Text 1.2: Mentor Farmer Interview Guide, Round 2…….…………………………………...3

Supplemental Figure 1: Study Flow,,,,,,,,,,,,……………………………………………………………...…………4

Supplemental Figure 2: Trajectory Analysis………………………………………………………...…………4

Supplementary Data

1 SUPPLEMENTARY TEXT 1: MENTOR FARMER INTERVIEW GUIDE

Each interview began with the interviewer introducing themselves, reminding the mentor farmer that their participation in the interview is voluntary, emphasizing that there is no right or wrong answer, and reminding them that their responses will only be shared with researchers.

1.1 **Round 1 Interview.** The interview guide used during the in-depth interviews of mentor farmers in July/August 2016, right as the intervention began.

1. Tell me everything that has happened in SNAP since we last talked in December
   [*Probes for each of the following events:* What happened? What do you like/not like about it?]
   a. Annual planning meeting (January)
   b. Survey (January)
   c. Mentor Farmer meeting (March)
   d. Dr. Mbwaga’s visit and Mr. Makenge (March)
   e. Visit from Dak and Aster (April)
   f. Dr. Mbwaga and Mr. Makenge, second visit (May)
   g. Harvest data collection
   h. Mentor Farmer meeting (June)
   i. Graph discussion (May/June)
   j. Other events or conflicts, such as people leaving/joining the project or especially difficult people
2. Have you stopped holding meetings? Why/why not? From when to when?
3. How do you feel like this project is going?
4. This intervention is participatory. Can you tell me what that means?
5. What in the village needs more work?
6. How is working with your peer mentor farmer?
7. What is your plan for the group in the future?

1.2 Round 2 Interview. The interview guide used during the in-depth interviews of mentor farmers in November/December 2016, after the first semester of the intervention.

1. Can you describe your experience in SNAP-Tz so far / since the last time we spoke?
    a. What did you do? What happens? How do you feel about that?
    b. Are there any specific success stories you want to share with us?
    c. Are there any specific challenging experiences you want to share with us?
2. One woman and one man is chosen as mentor farmers in each village.
    a. What do you think about this?
    b. In what ways do you think your roles will be different? In what ways will they be the same
    c. As a wo/man, what advantages did you experience so far compared to the other mentor farmer?
        i. As a wo/man, do you think you will have in the future compared to the other mentor farmer? Any topic that will be easier for you to approach?
    d. As a wo/man, what challenge did you experience so far compared to the other mentor farmer?
        i. As a wo/man, what additional challenges do you think you will have compared to the other mentor farmer? Any topic that will be harder for you to approach?
3. What messages about men and women have your learned in SNAP?
    a. Who is the primary decision maker?
        i. How much input do other members of household has?
        ii. Why is this the case?
    b. Are there instances when you and your spouse disagree?
        i. Can you please tell me about it?
        ii. How did you solve the disagreement?
4. Is there anything else we should know?

2 SUPPLEMENTARY FIGURE 1: STUDY FLOW

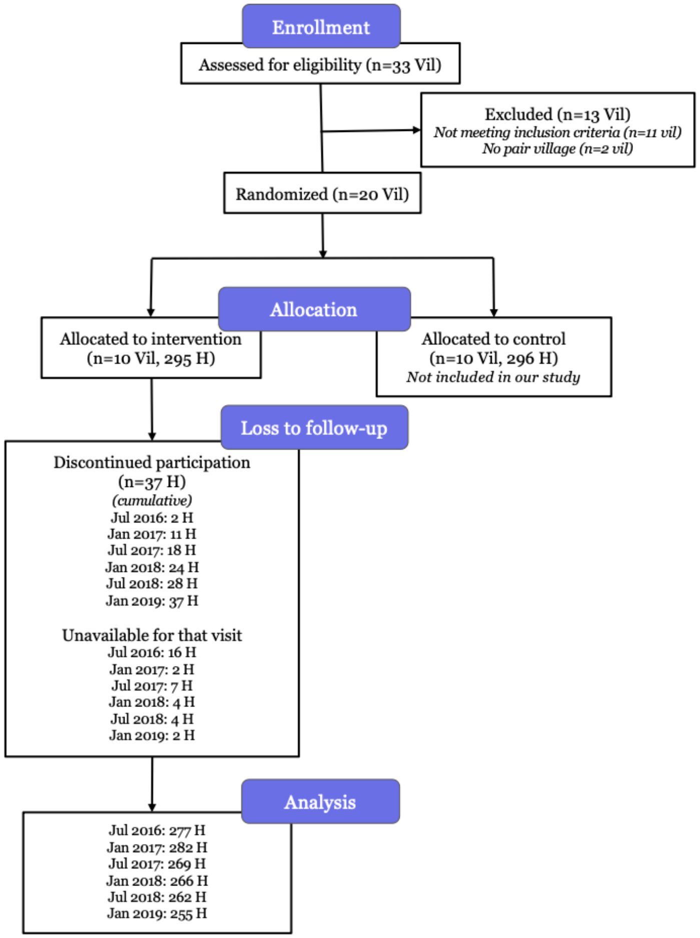

Supplemental Fig. 1: Participant flow for our participation analysis using the parent study, the Singida Nutrition and Agroecology Project (SNAP-Tz). Abbreviations: Vil: village, H: household

2 SUPPLEMENTARY FIGURE 2: TRAJECTORY ANALYSIS

A. B.

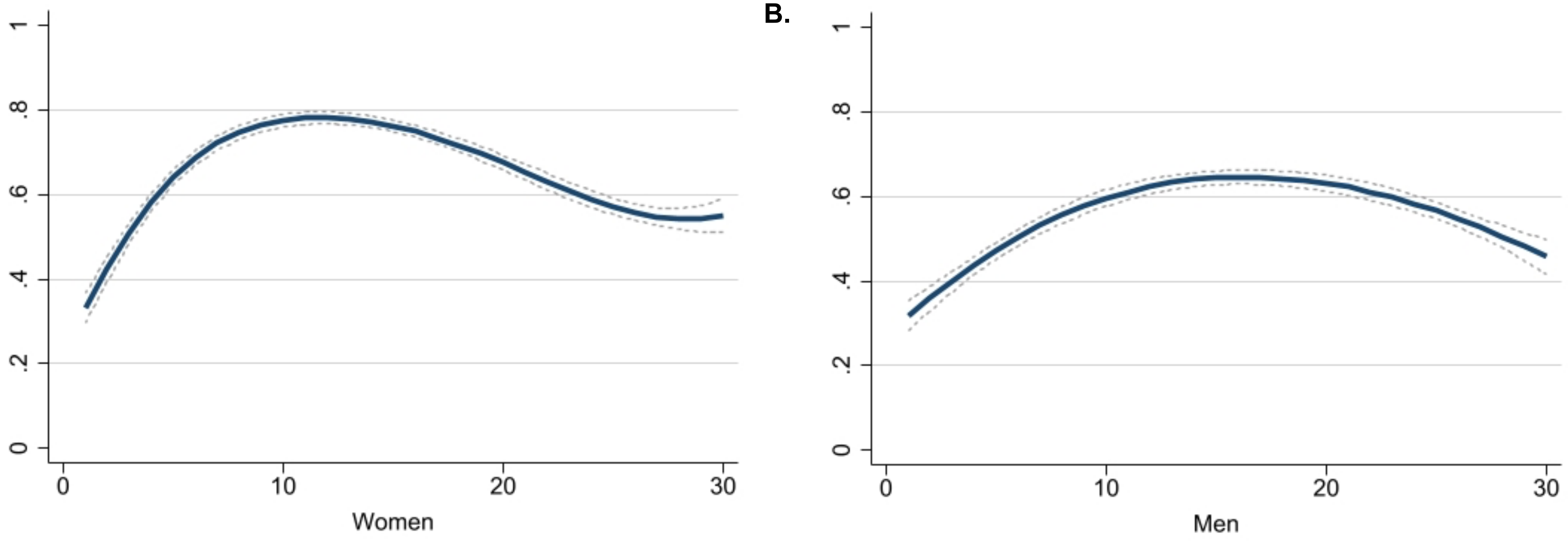

Supplementary Figure 2: Trajectory analysis of participation intensity of Singida Nutrition and Agroecology Project's participants revealed one latent trajectory group.
